# Contraceptives Are Also Drugs

**DOI:** 10.7759/cureus.35563

**Published:** 2023-02-27

**Authors:** Satish Jankie, Sameera Sampath, Lexley M Pinto Pereira

**Affiliations:** 1 School of Pharmacy, The University of the West Indies, St. Augustine Campus, TTO; 2 General Medicine, HealthNet Caribbean, Cunupia, TTO; 3 Department of Pharmacology, The University of the West Indies, St. Augustine Campus, TTO

**Keywords:** quality in health care, general medicine pharmacology, education and training, clinical pharmacology, patient education, lifestyle drugs

## Abstract

We report a patient who stated that contraceptives are not drugs. She presented with distressing symptoms of UTI following sexual activity and denied using any medication. Her physician prescribed co-amoxiclav based on her urine culture and sensitivity report, and the patient returned three days later with complete relief of symptoms but complained of vaginal bleeding. The patient then disclosed that her gynaecologist administered a contraceptive injection one month prior for endometriosis. When asked why she did not disclose this information at her previous visit, she responded, “that’s* not a drug, it is a contraceptive*.” It is essential to inquire from every woman of childbearing potential if she is currently using contraceptives to enhance patient care and for public health considerations.

## Introduction

In 2019, of the 1.9 billion women between the reproductive ages of 15 and 49 years, 1.1 billion required family planning services, and 842 million were using a modern method of contraception. More than 151 million (16%) women were using oral contraceptive pills (OCPs) globally, and more than 20% of women in 27 countries preferred this method of contraception [1]. The efficacy of OCPs may succumb to the issue of missing a pill, reducing the therapeutic effectiveness of these agents. Injectable contraceptives containing progestogen like medroxyprogesterone acetate (DMPA) provide a viable option for breastfeeding women and those unable to use estrogen-based pills. DMPA use circumvents the necessity for swallowing a tablet at the same time daily and enhances its use as a contraceptive for pregnancy prevention. The indications for these agents extend beyond contraception to regulating irregular cycles and endometriosis and providing relief from dysmenorrhea and menorrhagia. Progestogens with anti-androgenic properties have been found to control seborrhoea, acne, hirsutism, and alopecia [2]. The benefits of pharmacological contraception include prevention/delayed development of pelvic inflammatory disease, rheumatoid arthritis, and menstrual migraine. It improves bone mineral density and reduces the risk of endometrial, ovarian, and colon cancers [2]. These agents are included in the WHO list of essential medicines, as they meet the priority healthcare needs of the population [3]. The young woman we reported plainly believed she was receiving a means to stop the discomfort of endometriosis and was shocked to learn that a contraceptive is a drug.

## Case presentation

A 28-year-old female presented with painful and troubling symptoms of frequent micturition, burning, and dysuria, following recent sexual activity; this was the second occurrence within the month. She denied using any other medication, and based on her clinical presentation and culture-sensitivity report of a heavy growth of coagulase-negative Staphylococcus, amoxicillin/clavulanic acid was recommended. She was advised to hydrate frequently, alerted to possible vaginal candidiasis, rash, and diarrhoea, and requested to return for a follow-up visit after seven days. 

After three days, the patient returned with complete relief of lower urinary tract symptoms but now complained of vaginal bleeding, which she staunchly attributed to the antibiotic. She denied using any medication on her previous visit. Still, when directly questioned about any gynaecological issues or treatment she may be receiving, she admitted her gynaecologist had administered an injection of DMPA 150 mg via the intramuscular route one month earlier for endometriosis. She stated that this was not disclosed on questioning at the earlier visit because she believed that “it is not a drug, it’s a contraceptive.” She was counselled, reassured, prescribed ibuprofen, and told to visit her gynaecologist.

## Discussion

OCPs can be acquired without a prescription at community pharmacies in Trinidad and Tobago. Adverse effects may be mild, such as nausea, oedema, and headache, to moderate symptoms of breakthrough bleeding, amenorrhea, and weight gain. OCPs are associated with severe adverse effects, such as dyslipidaemia, venous thromboembolic disease, and cholestatic jaundice. The severity of these adverse drug reactions highlights the necessity for adequate patient education so they can report to their physician if necessary. Interactions with antimicrobial agents can reduce the efficacy of oestrogen-containing agents, as can inducers of the hepatic microsomal metabolizing enzymes like rifampicin. Contraceptive counselling must extend beyond compliance to include unwanted effects, and patients should be informed when medical attention is required. DMPA is a reversible progestogen-containing contraceptive that inhibits ovulation by suppressing the pituitary release of follicle-stimulating hormone and luteinizing hormone. It is propitious to women >35 years old who smoke and have migraine headaches, endometriosis, or sickle cell disease. Its therapeutic versatility extends to women with a history of thromboembolic disease or patients having difficulty complying with other methods of contraception/contraindications to oestrogen use [4]. To our knowledge, there is no reported drug-drug interaction between co-amoxiclav and medroxyprogesterone acetate, which undergoes glucuronidation by CYP3A4 while co-amoxiclav is excreted unchanged in the glomerular filtrate. DMPA may produce a thin and atrophic endometrium, which can precipitate irregular, unpredictable bleeding/spotting, especially in the first few months of use [5]. The prevalence of irregular bleeding due to DMPA is associated with the duration of its use. Initially, irregular bleeding may occur at unpredictable intervals, but the likelihood that it may occur decreases with the increasing number of injections given.

Contraceptives received a marketing thrust in the 1990s by being promoted as “lifestyle drugs” because beyond fertility control, they could address various conditions like acne, moods, and menstrual irregularities and even delay/postpone menstrual bleeding to permit social obligations. Lifestyle drugs “improve a person's quality of life by treating less serious conditions; also called cosmetic, life-enhancing, recreational, or discretionary” [6]. Their easy availability and aggressive marketing for “non-medical/paramedical” conditions may prompt consumers not to perceive these agents as drugs.

Through her belief that an injectable contraceptive is not a drug, this patient failed to check with her gynaecologist and presented with an avoidable scenario of breakthrough bleeding. Given the causality assessment of this drug, there is an increased likelihood of irregular bleeding during the initial phases of therapy. This case highlights the requirement for health literacy for patients and signifies the role of healthcare providers in advising patients who use these preparations. This patient was oblivious to breakthrough bleeding as an adverse effect of the injectable contraceptive and erroneously attributed it to amoxicillin/clavulanic acid. While considering sensitivity and privacy in questioning patients about the use of these drugs, clinicians should elicit such drug history in women of reproductive age. Patient education on the range of adverse effects from mild to severe should be encouraged at both the prescriber’s office and the pharmacy. Physicians should also assess women for medical eligibility before and during the use of hormonal contraceptives. Additionally, there is a need to document the patient’s medication history, which should be made available to clinical pharmacists. Consultation with a pharmacist to ensure the safety and compatibility of co-administered medications is essential for patient safety. A pharmacist’s assessment of the medication history of a patient can result in patient counselling to increase awareness of the possible adverse effects of the patient's prescription.

## Conclusions

We believe it is essential to enquire from every woman of childbearing potential if she is using any contraceptive therapy to elucidate clear guidance for the safe and effective use of contraceptive agents. Women should be alerted to their unwanted effects, and clinicians should discuss the same when prescribing these drugs to women of childbearing age.
